# A Critical Discourse Analysis of an Australian Incarcerated Trans Woman’s Letters of Complaint and Self-Advocacy

**DOI:** 10.1111/etho.12343

**Published:** 2022-06-21

**Authors:** Sherree D. Halliwell, Carol du Plessis, Andrew Hickey, Jessica Gildersleeve, Amy B. Mullens, Tait Sanders, Kirsty A. Clark, Jaclyn M. W. Hughto, Joseph Debattista, Tania M. Phillips, Kirstie Daken, Annette Brömdal

**Affiliations:** School of Creative Arts at the University of Southern Queensland; School of Psychology and Wellbeing at the University of Southern Queensland; Communications in the School of Humanities and Communication and Chair of the Human Research Ethics Committee at the University of Southern Queensland; English literature at the University of Southern Queensland; School of Psychology and Wellbeing at the University of Southern Queensland; School of Psychology and Wellbeing at the University of Southern Queensland; Department of Medicine, Health, and Society and Program in Public Policy Studies; Brown School of Public Health and faculty in the Center for Health Promotion and Health Equity at Brown University; (Metro North Health) based at the Metro North Public Health Unit; School of Psychology and Wellbeing at the University of Southern Queensland; Centre for Health Research, Institute of Resilient Regions, University of Southern Queensland; School of Education

## Abstract

This case study provides a critical discourse analysis of 121 letters of complaint and self-advocacy authored by Natasha Keating, a trans woman incarcerated in two Australian male correctional facilities from 2000 to 2007. During her incarceration, Natasha experienced victimization, misgendering, microaggression, and institutional discrimination. Despite this, Natasha embodied and “fought” against the injustices she experienced, whilst seeking to speak for other trans incarcerated persons also silenced and treated with indifference, contributing to changes in the carceral system. This original case study analyzes the discursive strategies Natasha employed to construct and reclaim an affirming self-identity through a deliberate campaign to effect social change and policy concessions within a system designed to curtail self-determination. Through her empathic and impassioned letter-writing approach, leveraging a *military metaphor*, this novel analysis showcases the significant implications her activism/agentism and determination had in naming and seeking to dismantle the systems of oppression trans incarcerated women experience.

## Introduction

This case study examines an archive of letters written by Natasha Keating, a trans woman who was incarcerated in two male correctional facilities in Queensland, Australia from 2000 to 2007 (aged 23–30 years). During her incarceration, Natasha experienced victimization, misgendering, microaggression, and institutional discrimination. Despite these hardships, Natasha wrote over one hundred letters of complaint to both authorities and personal contacts advocating for her own health and rights, and that of other trans incarcerated persons. She sought access to gender affirming supports and care, clothing, grooming, hygiene, and commissary items (including female undergarments) and suitable housing assignment in line with her heightened vulnerability. Natasha’s letter writing paved the way for important future policy and procedure revisions within the carceral system for trans persons in Queensland, Australia.

This article is written to honor Natasha and seeks to showcase how, despite experiences of victimization, she advocated fiercely for herself (and ultimately for others) whilst incarcerated. The adoption of a discourse-historical approach (DHA) to critical discourse analysis (CDA) (Wodak et al. 1999, 14) provides the opportunity to examine Natasha’s lived experiences through these letters and related correspondence (Garcia 2016; Merriam and Tisdell 2009). The value of Natasha’s letters rests with the insights they provide into daily life and the challenges she encountered in seeking recognition. As Angela Garcia (2016) notes, archives of letters from and to incarcerated persons provide an insight into “both the rhythms of daily living and the longue durée” (575) of the prison term. Natasha’s letters provide what Garcia (2016) identifies as “a tactile sense of history and sociality” (578) where affective connections to the experience are writ-through the archive of letters. It was from this perspective that the discourse analysis reported in this paper was broached, with the letters providing more than “data,” but “a genre of living that is at once unique … and also illuminates the realities” of prison life (Garcia 2016, 578). Further, Natasha’s letters also demonstrate how she utilized letter writing as an instrument of change to both effect policy concessions and enact an aspirational identity as an activist and agent of social justice to better inform gender-affirming carceral strategies and policies. This study focuses on how Natasha leveraged a deliberately framed discourse – one we cast here under the guise of *military metaphor* – to define her self-identity (Opsal 2011). Through this, she was able to subvert the homogenizing effects of the prison as a “total institution” (Goffman 1961, 4), an institution overtly designed to produce a compliant and uncomplicated population through the “stripping” of inhabitants’ identities and senses of Self.

Research from the US and Australia suggest that trans incarcerated persons, especially trans women, are a “vulnerable group” (Brömdal et al. 2019; Brömdal et al. 2022; Brown 2014; Lynch and Bartels 2017; U.S. Department of Justice 2012) who experience significant and disproportionate violence, harassment, and sexual mistreatment by other incarcerated persons and correctional staff due to having a gender identity or expression that does not align with socially constructed norms, rooted in cisnormativity and heteronormativity (Brömdal et al. 2019; Jenness and Fenstermaker 2016; Lynch and Bartels 2017; National Center for Transgender Equality 2018; Phillips et al. 2020; Rosenberg and Oswin 2015; Brömdal et al. 2022; Clark et al. 2022; Hughto et al. 2022; Stanley and Smith 2015; Van Hout, Kewley, and Hillis 2020). Moreover, within correctional settings, trans women often have limited access to gender-affirming medical care and a lack of gender-affirming accommodations (Brömdal et al. 2019; Brown 2014; Van Hout, Kewley, and Hillis 2020; White Hughto et al. 2018; Clark, White Hughto, and Pachankis 2017; Brömdal et al. 2022; Hughto et al. 2022).

The significant harms experienced by trans women within the male carceral system are well documented by the literature; however, McCorkel (1998) has shown that when institutional identity claims are excessively inaccurate or degrading, inhabitants are often jarred into defending their self-identity by generating idiosyncratic and resistant modes of self-expression. Resistance toward institutional strategies of control emerges through these individual constructions of alternative narratives of Self, particularly when focused on the expression of an “authentic self” (see Maruna 2001; McAdams and Bowman 2001; McCorkel 1998; Opsal 2011; Snow and Anderson 1987).

The project of identity formation and assertion within the context of the prison is “profoundly complicated” (Erzikova, Mills, and Sparks 2014, 143), for all incarcerated persons owing to a poverty of agentic choices within a system designed to homogenize its inhabitants. Self-narrative within the carceral system is bound by the constraints inherent in the system (Hardie-Bick 2018; Sanders et al. 2022) and incarcerated persons face the task of constructing self-narratives that allow for survival within hostile and dehumanizing environments. For this reason, self-narratives within this context are frequently pessimistic and self-condemning (Hardie-Bick 2018). Such a context leaves inhabitants who contravene normative identity categories vulnerable to stigmatization as social deviants, and following Goffman (1961, 1963), we note that total institutions such as prisons, mental institutions, and boot camps function as “forcing houses for changing persons” (1961, 12), the overarching purpose of which is to reshape their subjects as compliant and socially acceptable citizens. This strategy of control inhibits the possibility for demonstrating self-determination and places restrictions on physical bodies with consequences for incarcerated trans women.

We utilize CDA to analyze a corpus of 121 letters of complaint and self-advocacy authored by Natasha and explore the discursive strategies she employed to construct an affirming self-identity through a deliberate campaign to effect social change within the carceral system. We acknowledge the scholarship of other trans scholars, activists, advocates, and prison abolitionists that have similarly sought to campaign to effect social change (Stanley and Smith 2015;Stanley, Spade, and Queer (In)Justice 2012). We diverge from prior scholarship published in *Ethos* in focusing our research within a carceral environment where demonstrating self-identity is largely curtailed. We present a deep understanding of how Natasha utilized letter writing as an opportunity for the expression of an “authentic self” (Andrews, Clark, and Baird 1997). The narrative strategies deployed by Natasha demonstrate how an assertion of identity was made possible and how letter writing opened opportunities to chronicle her experiences as a trans woman. Through mastery of the legal instruments of carceral governance and constructing a heroic *warrior metaphor* narrative, Natasha challenged the regulating force of the total institution to the point that she successfully rallied community support to her cause. This method of creating “vivid versions of personal identities by challenging existing identities” allowed Natasha to “contest or resist a stigmatized identity” by asserting her own sense of Self (Opsal 2011, 138). This novel analysis of the discursive strategies used by Natasha will highlight how power can be challenged and how an affirming sense of Self can emerge, even within the context of a total institution (Goffman 1961). The case showcases the significant implications agentism and determination can have in naming and seeking to dismantle the systems of oppression trans women experience while incarcerated.

## Theoretical Framework

This paper applies a CDA approach informed by the field of Queer Linguistics. CDA aims to reveal the sources of power, dominance, inequality, and bias within written and spoken discourse (see Van Leeuwen 1993; Van Dijk, 1997, 2001; Wodak 2006). Ruth Wodak articulates that CDA’s central aim is “to investigate critically social inequality as it is expressed, constituted, and legitimized by language use” (2006, 53). Extending this orientation, Taylor (2010) points out that CDA is focused on “the study of how meanings are established, used, challenged and changed (including in talk)” (np). By considering the specific language and discursive tactics used by groups to dominate others, and how these discursive tactics perpetuate and legitimate ideologies and systems of governance, CDA exposes how discourses as *ways of speaking and thinking about the world* centralize power, define normative conceptions of the world and peoples and marginalize subaltern “Others” (Fairclough 2005; Rogers et al. 2005). CDA is therefore particularly relevant to historical case studies such as this, which draw from an archive of multimodal data spanning several years and are set within repressive institutional contexts. However, as Masoumeh Karimi and Hossein Tabrizi (2015) and Mongie (2016) argue, comparatively few CDA studies have focused on everyday discourse between individuals or on revealing how those in marginalized positions have employed counter-discourses to convey alternative views, challenge authority, mobilize support for their ideologies, and effect change. Mongie (2016) also notes that few studies have applied a CDA approach specifically to LGBTQI liberation discourse (cf. Baker 2005, 2006, 2008, cited in Motschenbacher 2011, 166) or have challenged the dominant heteronormative and cisnormative narrative constructed by institutions. This study sought to address this lacuna by using Natasha’s accounts of her experiences within the carceral system in Australia to expand the purview of CDA scholarship. By demonstrating the utility of its approach in highlighting the conflict between powerful discourses and the enactment of individual agency, the analysis of Natasha’s accounts provides an opportunity for expanding applications of CDA to the affirmation of trans self-identity.

The research from which this paper reports firstly sought to make explicit the relationship between language, gender, sexuality, and agency inherent to Natasha’s accounts, revealing the political motivations that drove her narrative reflections and discursive framing of her experiences. We draw on Goffman’s (1974) concept of “framing” wherein actors “select some aspects of a perceived reality and make them more salient in a communicating text, in such a way as to promote a particular problem definition, causal interpretation, moral evaluation, and/or treatment recommendation” (Entman 1993, 52). This analysis revealed how Natasha set about to not only frame (and provide a sense of legitimacy for) an account of her own experiences, but also establish counter-frames that challenged the legitimacy of the dominant, heteronormative and cisnormative institutional narratives she confronted.

Extending this preliminary focus, the analysis of Natasha’s accounts prompted a secondary objective to reveal the *constructive strategies* Natasha used to narrate her identity. Central to Wodak and colleagues’ (1999) approaches to CDA is the concept of the “narrated self” (15) whereby “[n]arrative identity allows various different, partly contradictory circumstances and experiences to be integrated into a coherent temporal structure, thus making it possible to sketch a person’s identity against the background of a dynamic constancy model which does justice to the coherence of a human life” (14). By identifying the “contradictory circumstances” inherent to Natasha’s accounts, insights emerged into the way she set about affirming a sense of Self through her experience of prison life and encounters with the total institution. Following Clary-Lemon (2010), we adopted an approach that focused on identifying the linguistic strategies employed by Natasha to construct a sense of an aspirational Self, and from this, we found an identifiable corpus of language and discursive strategy: we recognize a persistent *military metaphor* within which Natasha assumed a *warrioress* persona. Natasha was prone to describing her experiences (understandably enough) in terms of conflict, assault, and trauma, and we make use of *military metaphor* to illustrate the way she set about discursively framing her accounts.

As Steinert (2003) explains, military metaphor is hegemonic in many twentieth-century lexicons, having “invaded (!) the public discourse in economics, international relations, sport, even some medical specialities—and, of course, crime policy and policing” (266). Used internally by those facing significant peril, military metaphor is omnipresent where humans encounter challenge, resistance, or threat (Steinert 2003). Military metaphors such as “battling” or “fighting” the “enemy” are dominant within medicine, in particular oncology and HIV management (Semino et al. 2018; Sontag 1979). Framed as a universal call to action, military metaphor has driven public awareness, helped to minimize the spread of HIV, and, as described by Jing-Bao Nie and colleagues (2016), prompted “whole societies to mobilize, human, economic and social resources for health care and medical research” (3). At the individual level of suffering, Tate and Robert Pearlman (2016) note that use of military metaphor within treatment regimens can empower patients by evoking a sense of determination and inner strength. However, Shapiro (2018) argues, when patients internalize the war and come to embody the battle ground itself, this can lead to depression as they courageously fight down feelings of distress and despair. While a growing body of clinicians and academics advocate for the retirement of the military metaphor within medicine (see Nie et al. 2016; Shapiro 2018; Tate and Pearlman 2016; Hendricks et al. 2018), the emergence of COVID-19 as a global threat has rather reinforced the supremacy of military metaphor in how the medical profession, communities, and governments reference and respond to universal health threats (Gillis 2020).

In the public arena, military metaphors are particularly prevalent within social work, crime, and policing due to the inherently adversarial nature of these fields. However, much of the scholarly literature pertains to the policies and practices employed by public institutions, rather than exploring the experiences of those affected (Beckett 2003). Within correctional institutions, incarcerated persons are denied freedom at a structural level by the system and at a personal level by those corrections officers that enforce and maintain order. Besides these structural tensions, microaggressions in denials of requests can be perceived as attacks on individuals’ rights and liberties, which often go unseen by the wider society outside the institutions’ walls. As Chris Beckett suggests, “in order to articulate and ‘make visible’ these experiences we naturally turn to the most visible form of human conflict—war” (2003, 637). This case study aims to make visible Natasha’s experiences by examining her use of military metaphor within the incarnation context.

## Methodology

### Biographical Context

Natasha Keating was born in a regional city in Victoria, Australia, in 1977, and from an early age sought to emulate women including the popular singer and actress Madonna in ways which did not align with normative social expectations. Although Natasha’s mother was supportive as she attempted to navigate her gender dysphoria, she was a target of bullying and victimization at school to the extent that she left formal education at 13 years of age. In her mid-teens, Natasha became involved in illicit drug use and sex work, and quickly became known to the police for shoplifting and other minor offences (Australian Transgender Support Association of Queensland [ATSAQ] 2008, 2; ATSAQ personal communication 2019, 2020). This illicit, drug-related offending escalated to an armed robbery in 2000 for which she served 7 years in two men’s prisons in Queensland, Australia. During this timeframe, she wrote the letters in focus here, a period when the carceral system within which Natasha was housed adhered to no formalized strategies, policies, or management procedures to appropriately support trans persons. On her release, Natasha moved home under the care of her mother and stepfather. Although her mother thought that Natasha’s mental health was improving, in August 2008, she found her daughter passed away, in her bed in the family home (ATSAQ 2008, 2; ATSAQ personal communication 2019, 2020). Consistent with Natasha’s wishes, ATSAQ gifted the documents to a library in Australia, where they remain publicly available and used here in accordance with the instructions Natasha left. Natasha had hoped that scholarly interest in her experiences would effect social change, and we have prepared this paper with this wish in mind.

### Data Set

The data set (see Table 1) comprises an archive of letters that Natasha wrote to, and received from, people within and outside of the law enforcement and criminal justice system in Australia, including correctional staff and managers, medical health practitioners, support organizations, lawyers, anti-discrimination representatives, and friends, during the 7 years of her incarceration. Natasha made duplicates of all her correspondence with the authorities and enclosed these in her correspondence with ATSAQ, hence their inclusion in the Australian library’s archive. It also includes two national newspaper articles published and retained by Natasha in 2006. The data set was also prone to ethical clearance, with approval provided by the University of Southern Queensland’s Human Research Ethics Committee (H19REA236) to use Natasha’s real name and identifiable information in future publications. The decision to use Natasha’s real name was based on her desire to share her experiences and write an autobiography, which was expressed in the letters. This wish was further reinforced by ATSAQ and her mother, who corresponded with the research team (personal communication, January 2021) indicating that they believed that in order to recognize Natasha’s legacy, her name should be known. Consistent with Natasha’s wish to translate her lived incarceration experiences into an autobiography, this project seeks to honor her by shedding some light on this discourse:

I, Ms Shannon Keating, also known by the alias of “NATASHA” give you … The Australian Transgender Support Association of Queensland … full permission and release you of any liability to use my known alias of “NATASHA” for the purpose of the following: Use in the ATSAQ monthly newsletters; Interviews (oral/written/recorded/ videotaped/emailed); And/or anyway you see fit to better educate and inform people on the issues, facing transgender people and the transgender community. From this, the 13th day of February 2006 to the 13th day of February 3016.

In addition, organizations mentioned in the letters were approved by the University of Southern Queensland’s human research ethics body to be identified; however, to protect the privacy of other named individuals in the letters, they have been de-identified and anonymized.

Most of the archive is derived from the latter period of Natasha’s incarceration, spanning the 11 months from October 2005 to August 2006. Only four documents pre-date this time (three in February/March 2002 and one in November 2004). One document, a letter to ATSAQ, post-dates this period (March 2007). For this research, the letters were initially accessed from the library archive by the last author (AB) and were digitized, copied, and deidentified before sharing with the research team. These digital copies were then used as the basis of the analysis in this paper.

### Method of Analysis: DHA to CDA

The DHA to CDA (see Reisigl and Wodak 2009; Baker et al. 2008) offers a useful framework for uncovering the relationship between language and power extant within documents that span wide timeframes. The DHA proposes a four-level heuristic model that considers the “intertextual and interdiscursive relationships between utterances, texts, genres and discourses, as well as extra-linguistic social/sociological variables, the history and ‘archaeology’ of an organization, institutional frames of a specific context of a situation and processes of text-reception and text consumption” (Baker et al. 2008, 279–280).

At the first level, the analysis seeks to understand the underlying power relations and dynamics of influence and persuasion through the specific use of text-internal lexicon, phraseology, and metaphor. At this level, various *contextual* and *institutional* factors set the discourse within a social space and time, giving it enhanced meaning in terms of this contextualization (see Al-Momani 2014). The second level of analysis is specifically focused on interdiscursive and intertextual relationships, wherein the documents under analysis are placed in relation to other documents from which deeper meanings and verification can be established. The third level concerns the particular *situational* context in which the narrative is produced and consumed and includes a focus on those individuals responsible for the production of the communications core to the discourse. Finally, the fourth level calls for a broader analysis of the social, institutional, and political historical context and works back to the contextualization central to the first stage (Reisigl and Wodak 2009). In this final stage, emphasis is given to situating the document to establish its idiosyncrasy and peculiarity in context of a wider discursive field.

The DHA applied in this paper sought to identify the strategies Natasha used to legitimize, persuade, and effect change though her letter writing, with the focus of analysis applied against the four levels in the following way:

- First level: attention was given to defining the dynamics inherent to Natasha’s experiences as an incarcerated trans woman, her positionality as a trans identifying person within the prison system in the years 2000–2007, and the institutional context of this situatedness.
- Second level: focus was placed on the structure, word-selection, and epistemic authority characteristics inherent to Natasha’s letters of complaint.
- Third level: the identification of the lived reality of Natasha’s experience provided the focus of this level. The analysis was geared to constructing a sense of the ontological condition of Natasha’s experience and within which her letter writing activities could be contextualized.
- Fourth level: critique of the carceral system and the experiences of trans identifying people formed the focus of this level. At this level, commentary on the experience of incarcerated trans identifying peoples was formulated.

We proceed in the next section to frame the discourses represented in Natasha’s accounts against these levels and then turn to Natasha’s use of *perspectivization* (Reisigl and Wodak 2009, 94) to reconstruct an aspirational self-identity with the invocation of a military metaphor in her writing.

We acknowledge the limits inherent in the posthumous use of personal archives, as the noninteractive and nonreactive characteristics (Miller and Alvarado 2005) of such archives can make interpretation a concern (Ritchie and Spencer 1994). In a companion paper (. forthcoming), we move beyond this personal archive and explore Natasha’s advocacy from a broader perspective, incorporating interviews with her family and friends. In the companion paper, the role played by significant others in her resistance to the carceral system is explored in greater detail, while in the current paper, the attention is on the discourse used within the text of the archive.

Our readings of the archival data derive from multiple disciplinary and epistemic perspectives to enable a wide reading of this material and scope to the interpretative claims made in the analysis. In the first step of the CDA, the first and second authors reviewed the documents independently, ascertaining and verifying their chronology, removing duplicate correspondence, and assigning identified general codes to themes evident in the letters. This initial review process was inductive, and we used no predetermined codes. The first author then undertook a critical analysis of the documents, focusing on highlighting recurring codes, and from which a coding frame of short descriptors grouping similar ideas was produced (Owen 1984). These codes were inspected, collated, and reviewed by S. H., A. B., C. d. P., and A. M. and were used to confirm, reinterpret, and refine the analyses made of Natasha’s letters.

### Authors’ Positionality

This paper forms part of a larger body of scholarship focusing on the discriminatory and inhumane policies governing trans persons incarcerated in Australia and the US. Inspired by the scholarly work of trans scholars, activists, advocates, and prison abolitionists that have sought to campaign for social change within the carceral system (Stanley and Smith 2015; Stanley, Spade, and Queer (In)Justice 2012), the authors of this paper comprise a research team collectively committed to documenting the lived experiences of incarcerated trans persons and dismantling the oppressive policies still practiced in our carceral institutions. Our scholarship spans disciplines of gender and trans studies, sociology, clinical and health psychology, education, epidemiology, behavioral sciences, public health, medical anthropology, criminology, and critical policy analysis. The authors have been intimately engaged in trans rights and health research and activism and advocacy within the carceral system for 3–25 years and collectively have over 75 years of experience in the field. Our authorship team includes researchers of trans and cisgender lived and embodied experiences spanning sexual orientations (i.e., bisexual, pansexual, genderqueer, heterosexual) and includes both trans and cis persons with incarceration experiences.

## Findings

The analysis proceeds by considering the moral framing (and counter-framing) of being a trans person within the carceral system. This constitutes the first level of analysis. We were particularly interested in how Natasha identified the dominant institutional “convict” narrative and how her claims of a “moral high ground” situated a wider sense of the carceral context. She introduced the use of military metaphor to describe this context. From mid-way through her 7-year incarceration, Natasha became involved with ATSAQ, a state-specific trans support organization, which had a profound effect on Natasha, providing her with an external champion and ally. Encouraged by the support and validation that ATSAQ gave her, Natasha increasingly escalated her letters of complaint to higher authorities. Natasha’s letters demonstrate an evolution in her discursive strategies over time, highlighting her increasing sophistication, knowledge, and impatience for outcomes. Her passionate pursuit of justice and willingness to stand up for her rights are evident in these letters throughout her incarceration; however, she was increasingly emboldened to escalate her complaints to higher authorities as her knowledge and mastery of the legal instruments of change and her external support network grew. In the period from December 2005 to July 2006, Natasha constructed a more “righteous” tonality in her criticisms, founded on the legitimate exercise of her rights.

At this level of analysis, the military metaphor was used to construct a heroic sense of self-identity in the face of an intractable institution. This emerged as a dualism—cast in terms of a “battle” or “fight”—between Natasha and the bureaucracy of the carceral system. Notably, the metaphor gave specific meaning to Natasha’s sense of Self and how she saw herself as a combatant in this exchange. In these terms, the wider context was established: Natasha was set within a context that was combative and confrontational but within which she was determined to assert her own agency opposing a system that sought to deny her self-identity.

### Historical Legislative and Socio-Political Context

Included within the archive of letters are documents by agents of the Queensland Government’s Department of Corrective Services (DCS), which contextualize the legislative and policy environment during the latter stages of Natasha’s imprisonment (2005). In a letter dated June 22, 2005, the Acting Executive Director, Strategic Policy and Services of the Queensland Government DCS, writes to ATSAQ inviting the Brisbane Gender Clinic and ATSAQ to attend a roundtable discussion with officers of the DCS to discuss issues relating to the management of trans persons in prisons. An “Issues Paper” is enclosed with this letter, scoping the current legislative environment, management principles, and issues for discussion. The introduction to the Issues Paper notes that “[i]n Queensland there has been no integrated response to the identification and management of transgender offenders. In the past, DCS has managed transgender offenders on an as-needed individual basis” (np). Trans incarcerated persons at the time of Natasha’s incarceration were subject to and managed by two general instruments: the *Corrective Services Act 2000*, which recognizes “(a) the need to respect an offender’s dignity; and (b) the special needs of some offenders by taking into account … gender”; and the *Anti-Discrimination Act 1991* (amendment 2003), which makes it “unlawful to discriminate against a person on the basis of a person’s gender identity.”^1^

The Issues Paper acknowledges the rights of trans incarcerated persons to equitable access to all rehabilitative, educational, medical, safety, and external support provisions as “other prisoners”; however, it does not table for discussion any specific special treatment under these provisions. The roundtable’s “primary issue” for consideration is described as the “accommodation of transgender prisoners,” specifically whether trans incarcerated persons should be accommodated within a facility that matches “self-identification vs. legal identity vs. physical characteristics.” The paper goes on to specify its departmental procedure in relation to elective gender-reassignment surgery, which it states: “will not be undertaken during the period of incarceration” and the provision of hormone treatment, which “will only be provided to those transgender prisoners who have been receiving such treatment prior to being incarcerated.”

These accounts illustrate a legislative and policy environment that lacks specificity in its treatment of incarcerated trans persons. The principle of “equitable” access to provisions afforded to “other prisoners” fails to acknowledge the special and particular needs of trans incarcerated persons. In contrast with, for example, those identifying with specific faith and religious affiliations, no universally acknowledged policy designation existed in 2005 and 2006 to enshrine or enact trans-specific rights. Within this context of ambiguity, Natasha commenced writing.

### Moral Framing

Consideration of the macropropositions^2^ in Natasha’s letters of complaints reveals frequent allegations of injustice related to the denial of her trans rights. Natasha positions herself as the aggrieved party, framing her incarceration experience as unjust and negligent. In the following letter, dated October 21, 2005 to the General Manager of the prison within which she was incarcerated during this period, Natasha appeals to an epistemic authority to assert her lay knowledge of trans rights and a reasonable expectation of intervention:

Should you look into the matter seriously and speak with any qualified Gender Specialist, they themselves will tell you that it is important to my Mental and Emotional wellbeing to live as, and be treated as a woman … I do believe that this unreasonable refusal of a simple request was made without the proper insight into my situation, and the overwhelming emotional upset this and many other decisions over the past five years has caused is I think something that has to be looked at seriously.

“Diagnostic” framing is present in this excerpt, wherein Natasha diagnoses the problem as the “unreasonable refusal” of her request. The lexical term “unreasonable” constitutes the injustice component of the frame, which Natasha further diagnoses as a product of a lack of “proper insight” into “her situation.” “Prognostic” framing is also visible here, with which Natasha positions herself as the aggrieved party. The General Manager of the correctional center, as the recipient of the letter, is nominated as the actor who, in her view, ought to solve the problem. By appealing to the epistemic authority of “any qualified Gender Specialist,” she asserts a solution to the problem, that is to be “treated as a woman.” She then realizes motivational framing in her letters, constructing the negligence of decision makers as persistent and ignorant, suggesting that the impact of decisions taken over the preceding 5 years requires attention as “something that has to be looked at seriously.”

Natasha positioned her experiences of conflict, assault, and trauma as relating to a battle or fight between herself and the carceral system’s bureaucracy. She penned a “war ditty,” articulated in a letter dated December 14, 2005: “Never let anyone say that a good fight for the fight for good wasn’t a good fight indeed.” She also asserted in a newsletter her preparedness to keep fighting for her rights within the prison system through her writing:

Well here we go again boys and girls, the fight never ends! …. First were the bras, then the panties, and the battle for them was nowhere near the “BATTLE” I had for the size 9^3^’s but with a lot of help from my friends, we got them too! … and now we battle on, to get them to recognize that yes, I am a woman, hear me roar! …

(Natasha Keating, ATSAQ 2006)

Natasha’s discursive strategy is rationalized and positioned in relation to the context of her identity as a trans woman, or as Natasha described herself “I am a[n] M-F pre-op Transgender woman incarcerated in a men’s prison [and this does not give] anyone the right to treat me like I’m less of a human being” (Natasha, February 13, 2006). Her motivational framing in writing these newsletters was that they were intended to be read by the wider trans community and as such represent her sense of affiliation with a group of peers working toward a common goal. The newsletters also position Natasha as a positive role model, even while incarcerated, for others also fighting for recognition and social justice.

In another of her letters of complaint, this time to the Council for Civil Liberty dated December 30, 2005, Natasha explicitly frames her experiences as deliberately unjust and negligent. Where in the above excerpt Natasha explains the denial of her trans rights as proceeding from a lack of “proper insight,” in the next excerpt Natasha reports:

Issues pertaining to Medical & also the gross negligence when it comes to the Duty of Care that this centre has to me. (A Duty of Care that this centre has to all of its inmates) also the huge lack of Moral decency in their dealings with me.

This complaint presents a similar, morally framed macroproposition that the DCS has dealt with her unjustly, failing in its duty of care and acting immorally. Within this, “gross negligence” and “huge lack of Moral decency” constitute the injustice components of the diagnostic framing. However, in contrast to the unmodified lexicon “lack of” insight, Natasha uses the modifiers “gross” negligence and “huge lack” of human decency to emphasize the Department’s deliberate actions to deny her rights, suggesting the Department’s failure in their duty of care was both a choice and pathology. Prognostic framing is visible in the repetition of “Duty of care,” where Natasha explicitly uses the upper-case formation of “Duty” to emphasize the expected treatment of herself, both as an individual case and as one of a universal class of “all other inmates.”

Whereas Natasha appeals to the universal class in the above excerpt to claim her rights under a universal “duty of care,” in another letter of complaint to the General Manager of the Correctional Center on 20 December 2005, Natasha specifically contrasts her experience with other incarcerated persons with “special needs”:

I’m not asking to be put up on a pedestal because of my Gender, I’m just asking to be treated fairly … as when I look around this Correctional Centres well as other centre’s [sic] it is plainly visible that nationality and religions are catered to …To add to this Muslim diets and religion, Asian’s are catered to with certain food items and not last or least is everything that’s provided to [A]boriginal inmates including the [M]urri meeting place that was constructed here at [Correctional Center] … So I think we both know where I am heading with this and at the end of the [day] I’m just asking to receive fair treatment so I don’t have to go through the ongoing humiliation and discrimination.

This letter’s diagnostic framing is achieved through the repeated appeals to “be treated fairly” and “receive fair treatment.” The injustice component of the prognostic framing tasks is implied through the juxtaposition of Muslim, Asian, and Aboriginal incarcerated persons, whose needs are “catered to,” in contrast to the “discrimination” experienced by Natasha in the denial of her trans rights. Natasha’s rhetorical question, “I think we both know where I am heading with this,” implies that it is hypocritical of the Correctional Center to ignore trans rights if it recognizes those of religious, ethnic, and First Nations claimants.

The moral framing work of these earlier letters of complaint within the corpus (authored in 2005) demonstrate Natasha’s positionality as claimant to the “moral high ground.” Natasha reinforces this positionality in her letters throughout 2006 by increasingly citing legal statutes and policies to enforce the legitimacy of her claims.

### Mastery of the Instruments of Change

Over the 8-month period from January to August 2006, Natasha’s letters show an increasing complexity as she becomes an authority on her rights. She is proactive in procuring rules, precedents, and procedures, and judicious in citing sections and clauses from a range of federal laws, policies, and guidelines supporting her requests and complaints.

Natasha keeps abreast of current cases and developments in policy and case law pertaining to trans carceral treatment and rights. She often discusses new legal protections and standards in her correspondence with the Queensland trans support organization (17 January 2006): “did you see Friday’s paper – The Equal Treatment Bench Book for judges? It’s a big & very impressive step, & I have to say it … very progressive for the State of QLD [Queensland]!!” Natasha’s legal knowledge of the Corrective Services Act 2000 and the Anti-Discrimination Act 1991 is particularly sophisticated for someone of a nonlegal background and is evidence both of Natasha’s intellect and of the effort she expended to ensure that she understood the system that confined her.

In her July 2006 “Exceptional Circumstances” parole submission, Natasha submitted 312 pages of documentation in support of her application. In her response to the General Manager’s correspondence following a Section 38^4^ incident, Natasha corrects a reference to legal statutes:

Your reference to the CORRECTIVE SERVICES ACT 2000 specifically s.38 (2) (b) is incorrect, that particular reference is to the good order and security of the centre’, the section you were, I assume, looking for is s.38 (2) (a).

(Letter to General Manager, [Correctional Centre], 30 March 2006)

Between January and August 2006, Natasha cites a specific piece of legislature or Corrective Services policy in 13 of her letters (see Table 2). Natasha left formal education at the age of 13 years and did not receive legal training at any time prior to, or during incarceration. Her mastery of the legal instruments of change was self-taught using the resources at her disposal at the Corrections Centers where she was incarcerated. Through her early setbacks in claiming trans rights, Natasha became aware that moralization strategies and emphatic appeals were often ignored. Natasha’s use of newly acquired legal and policy knowledge was paired with increasingly assertive language. In earlier correspondence, Natasha constructs her complaint to the General Manager on October 21, 2005 using subservient modifiers: “Should you look into the matter seriously” … and “I do believe that….” In the correspondence to the General Manager of March 30, 2006, Natasha assumes the dominant position of instructor and educator, appealing to the epistemic authority of the relevant statutes to support her position.

We observe across her 2006 correspondence that Natasha maintains this approach to communication with external agents, using an authorization strategy to support her positioning. In the following response to the General Manager on February 16, 2006, Natasha asserts her right to abstain from the Correctional Center’s psychological support program: “I will not be attending these courses/programs and any recommendations for me to do these courses/programs will subsequently be met with a Judicial Review of the decision.” In her correspondence of March 24, 2006 to the Freedom of Information Officer at the same Correctional Center, Natasha asserts: “… please be advised that this type of behaviour will not be accepted, should I feel the need to involve a solicitor and have ALL my files subpoenaed, I will be more than happy to do so.” Through mastery of the legal instruments of change, Natasha positions herself as morally and intellectually superior to the carceral institution. She underlines her position in her March 20, 2006 letter to her support organization, ATSAQ, where she declares: “I’ll not lower myself to their level!!! … [staff] shouldn’t be in a position of power if they’re going to ABUSE that power and the trust that has been put in them…” Indeed, her letters ultimately effect a role reversal, going so far as to accuse the carceral system itself of criminality: “I want to reiterate the possibility and probability of CRIMINAL CHARGES for this type of interference with my mail” (Letter to General Manager, [Correctional Center], June 23, 2006). In addition to her assumption of the dominant moral and intellectual discourse prosody through mastery of the instruments of change, 2006 marked a change in Natasha’s perspectivization (Wodak 2009, 94) wherein she positioned herself as a “warrior” vis-à-vis the institutional “enemy.” Through the topos of appeal to authority (Wodak et al. 1999), Natasha was able to construct a righteous identity founded on the legitimate and “good” exercise of her rights.

### Mobilizing a “Heroic” Self-Identity through Military Metaphor

From December 2005 through to the end of her incarceration, Natasha uses military metaphors to construct a vision of her correctional experience as “war.” Use of metaphor in discourse highlights underlying thought processes that reveal how we see and respond to the world (Lakoff and Johnson 1980). Adopting Lakoff and Johnson’s (1980) framework, we suggest that the following underlying conceptual metaphors inform the use of military metaphor within Natasha’s discourse: “CORRECTIONAL FACILITIES ARE BATTLEGROUNDS” and “PRISON LIFE IS WAR.” These metaphors support Natasha’s subversion of the convict stigma (Goffman 1963), constructing a positive self-presentation and a framing of the carceral system as the negative other (Reisigl and Wodak 2001). She engages military metaphor to describe physical place, actors, and agents, the carceral system, and the actions taken by herself, correctional officers, institutional representatives, her support system, and the wider community. In letters to ATSAQ, Natasha uses fighting language on 22 occasions across 17 different letters of correspondence. As argued by Charteris-Black (2004, 92), “military metaphors rarely occur singly” and “typically cluster to produce a ‘battery’ of metaphors”; in Natasha’s correspondence, these recurring metaphors serve as a scaffold upon which she constructs her discourse of resistance. Within these discourses are three identifiable semantic categories that inform the ideational macropropositions (Fairclough 1995; Halliday and Matthiessen 2013) (see Table 3). First, she positions the carceral system as battleground; second, she assigns a binary adversarial relationship to its actors; and third, she ascribes moral legitimacy to her cause.

Natasha’s most frequent use of the metaphor refers to her engagement with the DCS as a “battle” or “war,” establishing a contextual framing of the carceral system as *adversarial*. The adversarial framing that Natasha applies to her relationship with the carceral system also mobilizes a force metaphor: “CONVICTION IS FORCE,” in which she describes her actions in fighting terms “STRIKES AGAIN,” “kick them down,” and “waging a war.” Thus, all actions taken by Natasha and agents of the Department are depicted as heightened, militarized, and *against* each other:

I could take on the Department of Corrective Services for them to allow me to have surgery … Although with the battle I’ve had just trying to purchase a pair of women’s runners^5^ and some basic toiletries, it would definitely be bigger than Ben Hur!

(Letter to Discrimination Lawyer, February 2, 2006)

Natasha’s framing of her relationship with the carceral system as adversarial also by implication calls for Natasha’s external support network to take sides, thus reinforcing the discourse of resistance. As argued by Nartey (2019), the articulation of an enemy performs the function of painting a target for one’s attacks, focusing one’s objectives and rallying solidarity around one’s cause. Letters to her trans support organization in 2006 increasingly feature the inclusive pronoun formation “we” rather than the singular “I” of earlier letters: “We’ve taken the beach at Normandy and France still lies up ahead!!!” (Letter to ATSAQ, January 30, 2006); and headlined in her letter of June 15, 2006: “WAR!!!!!! WE TAKE NO PRISONERS.” These calls for social action and unity in the face of a common enemy draw on historically embedded mental frames pertaining to war and conflict, within which the unrelenting, unjust, and ferocious enemy must be vanquished by a heroic defender. Upon hearing the appeal, it is morally incumbent upon the “good” to take up arms against the oppressor.

Natasha’s visualization of her incarceration as a “war” also creates a lived reality that reframes her punitive experience as a *righteous cause* to be fought against a tyrannical enemy, rather than a just punishment to be endured. The “(e)vilification” (Lazar and Lazar 2004, 236) of the carceral system juxtaposes the notions of good and evil, invoking “intense emotions of morality (i.e., right and wrong)” (Nartey 2019, 118). Lazar and Lazar (2004, 227) argue that the articulation of an outside enemy is “pivotal to defining, establishing, and maintaining a moral order.” Natasha characterizes her interactions with the DCS as a “good fight” in several letters, reaffirming her conceptualization of her actions as just and warranted. This lexicon becomes a mantra that Natasha uses to galvanize support and legitimize her claims: “take care and keep up the good fight! (I even have mum saying that now! GOD bless her)” (Letter to ATSAQ, May 15, 2006). By conceptualizing her struggles as a “good fight,” Natasha also reframes her individual experience as a symbolic cause that righteously demands universal participation in defending against the enemy. The attribution of “good” to herself and her supporters validates her actions, claims, and moral superiority; in contrast, the implication that the carceral system is unjust denies its actors their legitimacy.

We have shown that in correspondence with her trans support organization Natasha reframes her relationship with the carceral system as adversarial and seeks to mobilize ATSAQ’s external advocacy and support by describing her objectives as a morally just and universal cause. Natasha also mobilizes military metaphor to construct identities for herself and her supporters as “battlers” and “warriors,” thus enacting a strategy of positive self-presentation and negative other-presentation. In her letter of June 15, 2006, she fuels the discourse of resistance by combining a personification strategy with a call to action: “I am hoping to appeal to you both and your sense of ‘WARRIORESS-NESS.’” Natasha realizes the topoi of comparison and threat (Wodak et al. 1999), personifying herself as resilient, defiant, and just in her resistance, and contrasting this with the metaphor of a cowardly bully, as suggested in the following excerpts: “… hear me roar! … I will not be stood over” (un-dated February 2006); “…they’re a little bit too scared to attack me directly and so they should be!” (March 21, 2006); and “… they were hoping to ‘cripple’ me emotionally, but enough to stop me fighting them” (March 30, 2006). The strategy of invoking an attack and defense metaphor by describing the carceral system’s actions as “stood over,” “attack,” and “cripple” also emphasizes the need for fighters to take on an active role in defending the “good.”

## Conclusion

The analysis demonstrated how Natasha leveraged discourse to “wage a war” against an unjust and cisnormative system and gain personal agency and power. While CDA typically exposes dominant or privileged narratives, this case study highlights the utility of using discourse as a source of power in nonagentic situations designed to curtail self-determination. Analysis of Natasha’s archive indicates that she innovatively pursued several discursive strategies, including appeals to epistemic authority; knowledge of instruments of change; using the tools of the system to legitimize her position within it; and military lexicon, phraseology, and metaphor to galvanize support and reframe positioning.

In addition, the analysis highlights changes over time in the strategies Natasha used, suggesting an increasing ability to “fight” discursively against a dominant and cisnormative governing institution. These include escalating concerns through the “chain of command,” creating connections with agencies, support people, and organizations to create momentum and pressure, ensuring greater transparency and accountability in relation to her concerns, employing shame and incongruence as a catalyst for action, and demonstrating authority in her approach. This combination of strategies increased her chance to be taken seriously and elevated her position, both in the eyes of the carceral institution and her own self-presentation, from that of a prisoner to an informed colleague or equal who commands respect, responsiveness, and for her concerns to be taken seriously.

In an interview conducted by the first and second authors in early 2021 with ATSAQ’s President, Gina Mathers, and Secretary, Kristine (Krissy) Johnson, Gina explained that: “Natasha wrote so well and was so comical that eventually people had to give in.” As a direct result of Natasha’s self-advocacy, the General Manager and deputy managers of the prison in which Natasha was latterly incarcerated arranged a meeting with ATSAQ to better understand the lived experiences of trans incarcerated persons. According to Krissy, tangible reforms that were implemented as a result of Natasha’s advocacy and the corrective service’s consultations with ATSAQ include provisions for preoperative trans women to be housed in single cells or with other trans women, with dedicated shower facilities.

Further reforms to the management of trans incarcerated persons have been implemented in the years after Natasha’s incarceration. In 2008, a formal procedure granted access to hormone therapy for those who had commenced treatment prior to incarceration (Rodgers, Asquith, and Dwyer 2017). In 2016, a Deputy Commissioner Instruction (Queensland Corrective Services 2016) concerning trans and intersex carceral management procedures included a number of significant statements supporting the right of individuals to self-identify as trans and to be accepted, treated, and referred to as their identified gender; to be managed on an individualized case-by-case basis through a multidisciplinary team; to not be isolated from other incarcerated persons, work, or programs, as a default prisoner management position; to be able to purchase items to reflect their gender identity; to be provided with gender appropriate clothing, including underwear at the discretion of the General Manager; and for all trans management decisions and concerns to be communicated to the Deputy Commissioner. In June 2021, a revised Custodial Operation Practice Directive (Queensland Corrective Services 2021) in relation to trans incarcerated persons was released with a clear focus on respect for human rights, diversity, inclusion, and equality. Placement decisions for incarcerated trans persons were to be based on a range of factors including health advice and the social circumstances of the individual.

Natasha’s unrelenting pressure and persistence articulated through these letters stands in marked contrast to the usual presentation of incarcerated trans persons as nonagentic victims of the carceral system. This paper shows how an imposed identity can be resisted within total institutions through the construction of alternative social change narratives that are reimagined and reinforced through discursive strategies. Although this is not the first time that letters from incarcerated trans persons have been analyzed (see Brown 2014), what this paper offers is an in-depth analysis of the discursive strategies employed by a single individual in order to reclaim agency within the carceral system. Through her empathic and impassioned approach, Natasha both embodies and “fights” against the injustice in the carceral system, whilst also seeking to speak for others who are silenced and treated with indifference.

## Figures and Tables

**Table 1.** Documents analyzed

Document type	Number of letters sent by Natasha Keating	Number of letters received by Natasha Keating
Higher legal authority—Anti-Discrimination Commission/Council for Civil Liberties/QLD Ombudsman/Supreme Court	20	13
Australian Transgender support Association of Queensland—ATSAQ	32	1
Carceral service—Department of Corrective Services	18	7
Correctional Center General Managers	25	10
Legal correspondence with independent counsel	7	
Medical correspondence with independent physicians and authorities	3	2
Grievance particulars, incidents and write-ups	11	0
Request for policies and documents	4	1
Policy documents and notices	0	2
Newspaper articles and material intended for publication	1	3
Property seizure notices	0	2

**Table 2.** Legislative and policy instrument analyzed

Date of correspondence	Legislative/policy instrument
January 11, 2006	Schedule 3, Corrective Services Act 2000
February 2, 2006	Section 166, Anti-Discrimination Act 1991
February 2, 2006	Chapter: 3 Breaches and Offences. Part: 1, 87 {6} of the Corrective Services Act 2000
February 20, 2006	Corrections Services Policy
March 24, 2006	s11e and s44(1) of the Freedom of Information Act
March 29, 2006	Corrective Services Act 2000, 30
March 30, 2006	Corrective Services Act 2000 s.38 (2)
May 25, 2006	Section 32DA of the Acts Interpretation Act 1954
June 13, 2006	Section 32(1) of the Judicial Review Act 1991
June 26, 2006	Chapter 5A Section 131 A ss (1)(a) & (b) of the Anti-Discrimination Act 1991 and s 7 ss (m) – gender identity
June 26, 2006	Section 159 (2) & (3) of the Anti-Discrimination Act 1991
June 26, 2006	Anti-Discrimination Act 1991 – s7 (M) Gender – Identity & s131A Vilification
July 14, 2006	Section 159 (2) & (3) of the Anti-Discrimination Act 1991
July 20, 2006	Section 7 (i) of the Anti-Discrimination Act 1991 – religious belief or activity
August 25, 2006	s 20 (2) (e), s 20 (2) (g) & s 20 (2) (h) Anti-Discrimination Act 1991

**Table 3.** Semantic categories, language, and ideational/interpersonal function

Date	Semantic category	Language	Ideational/interpersonal function
December 7, 2005	Carceral system	“Mammoth” batde	Adversarial
December 7, 2005	Values	good fight/fight for good	Righteous cause
December 14, 2005	Values	“War Ditty” Never let anyone say That a good fight for the “fight for good” Wasn’t a good fight Indeed	Righteous cause
January 17, 2006	Actions and agency	“Battle of the Bras”	Adversarial
January 24, 2006	Values	good fight	Righteous cause
January 30, 2006	Actions and agency	The batde hasn’t quite been won, and the war is really only beginning… We’ve taken the beach at Normandy and France still lies up ahead!!!	Adversarial
January 30, 2006	Action and agency	take the fight on	Adversarial
February 2,2006	Action and agency	batde I’ve had bigger than Ben Hur	Adversarial
February 20, 2006	Action and agency	have to batde have to batde	Adversarial
Undated (February 2006)	Action and agency	STRIKES AGAIN fight never ends the batde continues we batde on hear me roar! I will not be stood over we batde on the batde for them was nowhere near the BATTLE I had for…	Adversarial
March 30, 2006	Action and agency	for me to fight	Adversarial
	Values	they were hoping to “cripple” me emotionally, but enough to stop me fighting them	Righteous cause
March 21, 2006	Action and agency	they’re a little bit too scared to attack me directly and so they should be!	Adversarial
May 8, 2006	Adversarial system	taken up the WAR	Adversarial
May 8, 2006	Action and agency	fighting their B/S	Adversarial
	Action and agency	pick that battle back up off its backside and kick them down on theirs!!	Adversarial
	Carceral system	these battles and wars against the system	Adversarial
May 15,2006	Carceral system	another battle	Adversarial
	Action and agency	isn’t everything a battle!	Adversarial
	Values	feisty little battler	Adversarial
		keep up the good fight	Righteous cause
June 15, 2006	Action and agency	WAR!!!!!! WE TAKE NO PRISONERS	Adversarial
		I am hoping to appeal to you both and your sense of “WARRIORESS-NESS”	Adversarial
		waging a war	Adversarial
		wield so much power over so many lives	
June 17, 2006	Action and agency	battle for “pink & cute” runners	Adversarial

## Footnotes

1. Prior to 2003, “unlawful” discrimination “on the basis of a person’s gender identity” did not interpret “gender identity” to include trans persons—regardless of if they had affirmed gender surgically or legally. The 2003 amendment to the Anti-Discrimination Act 1991 interprets “gender identity” to include trans persons; however, trans persons are not *literally* specified or articulated in the Act’s interpretation of “gender identity” (ATSAQ personal communication, 2021).

2. Martin et al. (2018, 2) suggest macropropositions “are derived from the microstructure by deleting the unneeded propositions and generalizing some groups of propositions, forming the gist of the discourse” (Kintsch and Van Dijk, 1978).

3. Referring to a struggle to access a pair of pink women’s running shoes that took over 6 months.

4. Section 38 of the *Corrective Services Act 2000* (Qld) has four parts referring to special treatment orders that an authority may provide a prisoner special treatment. Part Two (2), mentioned by Natasha in her correspondence, has two components relating to when special treatment orders may be executed: part (a)—where it concerns “the prisoner’s safety,” or part (b)—“the security or good order of the corrective services facility.”

5. “Runners” is Australian slang referring to athletic shoes, sneakers, and running shoes.
